# CFD Analyses of Density Gradients under Conditions of Supersonic Flow at Low Pressures

**DOI:** 10.3390/s24185968

**Published:** 2024-09-14

**Authors:** Robert Bayer, Petr Bača, Jiří Maxa, Pavla Šabacká, Tomáš Binar, Petr Vyroubal

**Affiliations:** 1 Faculty of Electrical Engineering and Communication, Brno University of Technology, Technická 10, 616 00 Brno, Czech Republic; robert.bayer@vut.cz (R.B.); maxa@vutbr.cz (J.M.);; 2 Institute of Scientific Instruments of the CAS, Královopolská 147, 612 64 Brno, Czech Republic

**Keywords:** Ansys Fluent, ESEM, critical flow, CFD, nozzle, shock wave, Schlieren method

## Abstract

This paper deals with CFD analyses of the difference in the nature of the shock waves in supersonic flow under atmospheric pressure and pressure conditions at the boundary of continuum mechanics for electron microscopy. The first part describes the verification of the CFD analyses in combination with the experimental chamber results and the initial analyses using optical methods at low pressures on the boundary of continuum mechanics that were performed. The second part describes the analyses on an underexpanded nozzle performed to analyze the characteristics of normal shock waves in a pressure range from atmospheric pressure to pressures at the boundary of continuum mechanics. The results obtained by CFD modeling are prepared as a basis for the design of the planned experimental sensing of density gradients using optical methods, and for validation, the expected pressure and temperature courses from selected locations suitable for the placement of temperature and pressure sensors are prepared from the CFD analyses.

## 1. Introduction

This paper is part of a comprehensive research project on environmental scanning electron microscopy conducted by a team led by Vilém Neděla from the Institute of Scientific Instruments of the CAS in collaboration with the Department of Electrical and Electronic Technology, Faculty of Electrical Engineering and Communication. The environmental scanning electron microscope (ESEM) was developed to overcome the stringent vacuum requirements of traditional electron microscopy, enabling the observation of non-conductive, semiconducting [1,2], and native [3,4,5] samples without damage and facilitating in situ dynamic experiments [6,7,8]. The ESEM employs specialized ionization or scintillation detectors to detect signal electrons [9,10]. One aspect of this research focuses on supersonic flow in an aperture fitted with nozzle at low pressures, near the boundaries of continuum mechanics [11]. This phenomenon occurs in the differentially pumped chamber section of the ESEM, which separates two regions with a substantial pressure gradient in the order of approximately 2000 Pa to 70 Pa using an aperture. The nozzle’s shape and the controlled flow within this region significantly influence the scattering and losses in the primary electron beam passing through the differentially pumped chamber and the aperture with the nozzle, affecting the resulting image sharpness. Shock waves that arise in the supersonic flow region behind the aperture are a major factor influencing the beam scattering. Their type and shape can be controlled, among other factors, by adjusting the nozzle’s configuration, and these principles are well established under typical atmospheric conditions. However, in the given microscope, during its operation, we are operating near the boundary of continuum mechanics at low pressures, where the ratio of inertial and viscous forces is significantly different. This difference significantly impacts the formation and intensity of shock waves. At low pressure, due to the reduced density, the inertial forces are notably lower, while the viscous forces remain relatively independent of the pressure up to approximately 133 Pa, not decreasing with decreasing pressure.

**Figure 1:**
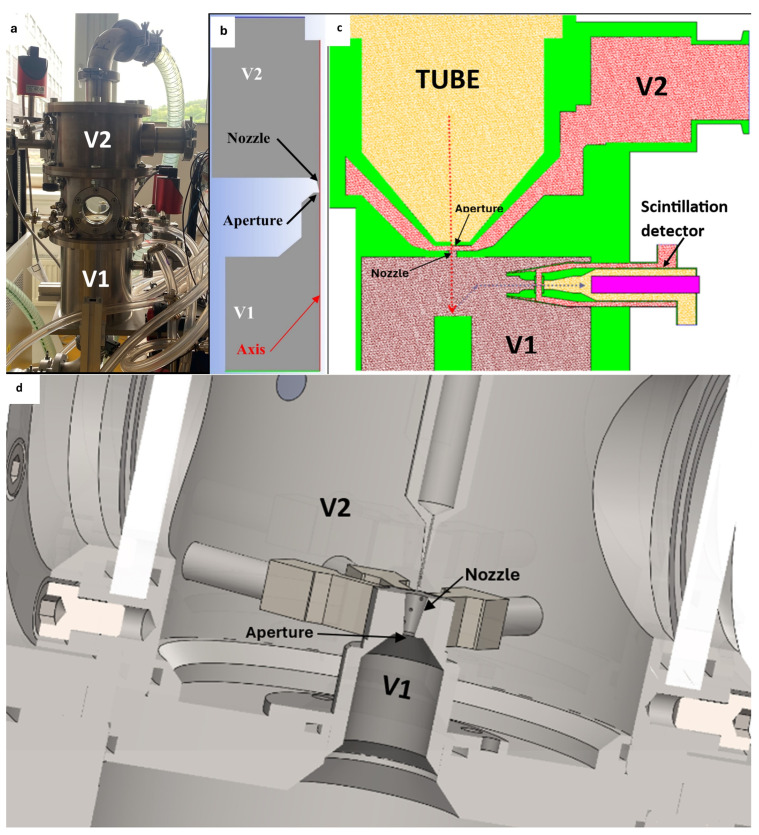
Real experimental chamber (**a**), simplified 2D axisymmetric model of the experimental chamber (**b**), scheme of the ESEM (**c**), and scheme of the experimental chamber (**d**).

For this reason, research is being conducted using, among other means, a multi-purpose experimental chamber (Figure 1a,b). This chamber simulates the operating conditions of the ESEM, a schematic of which is shown in Figure 1c. The chamber is modular, allowing for the exchange of various components, such as the nozzle shape (Figure 1d). Notably, the experimental chamber can be equipped with a variety of measuring devices and sensors that can be directly positioned within the flow axis, primarily for pressure and temperature measurements. To measure the pressures from the nozzle wall, small openings are located around its circumference. Additionally, optical measurement methods, such as the Schlieren technique, can be employed. For this purpose, the chamber is equipped with two windows (Figure 1a), allowing the flexibility to change the glass type based on the specific measurement requirements. The planned Schlieren experiment will enable the observation and characterization of shock waves generated under various pressure conditions, facilitating comparisons between low and atmospheric pressures.

The Schlieren method is an optical technique used to visualize density gradients within transparent media, thereby rendering visible phenomena such as heat, sound waves, and pressure variations. By exploiting the principle of refraction, even subtle differences in the refractive index can be detected and visualized. Common demonstrations include visualizing the thermal plumes from a candle flame or the propagation of sound waves through air. The method relies on precise optical components, such as lenses or parabolic mirrors, to detect deviations in light rays caused by refractive index gradients. Various configurations exist, including the Toepler system, Z-type system, single-mirror system, background-oriented Schlieren (BOS) system, and focusing Schlieren system. In the field of fluid dynamics, Schlieren imaging is an invaluable tool for detailed visualization of flow patterns, particularly in aerodynamic studies, where it is used to visualize complex phenomena such as shock waves. The materials studied using Schlieren techniques typically exhibit very weak refractive index gradients, making their visualization challenging but rewarding [12,13,14].

A key aspect of this research was to study and understand the issues presented by Dr. Danilatos in his works, as he is a pioneer of differential pumping in the ESEM [15]. In his work, including “Figure of Merit for Environmental SEM and its Implications” [16], Danilatos investigated the impact of the nozzle angle and thickness on the system performance in the absence of an aperture. His findings highlight the trade-off between a wider nozzle aperture (beneficial for electron beam transmission) and the vacuum system’s ability to maintain a sufficient pressure differential. A thinner nozzle facilitates electron beam passage, while a thicker nozzle supports better vacuum conditions. Danilatos further optimized the electron beam transmission based on density distribution in “Optimum beam transfer in the environmental scanning electron microscope” [17]. He determined that a thin nozzle minimizes the pressure barrier encountered by the electron beam. In “Beam transfer characteristics of a commercial environmental SEM and a low vacuum SEM” [18], he provided foundational insights into the density and pressure variations within gas flow through a small aperture separating chambers with a significant pressure differential. Using a ThermoFisher electron microscope (FEI) (Waltham, MA, USA) as a model, Danilatos explored the influence of the aperture size and controlled back pressure on the gas flow [18].

The first part of this paper presents experimental analyses of supersonic flow within a nozzle in an experimental chamber at a specified pressure gradient. The wall pressures were measured during these experiments. This serves as a validation step to fine-tune the CFD analysis within the Ansys Fluent system. Concurrently, initial calibration of the optical apparatus was conducted to acquire images of the first and second derivatives of the density values for the shock wave analysis. These results demonstrated the necessity of shortening the nozzle for further analyses, as some interesting shock wave results already appear in the nozzle itself. The second part of the paper presents CFD analyses as a foundation for future experiments. It explores the influence of varying pressure ratios on the location of shock waves. These analyses consider the impact of changes in the ratio of inertial and viscous forces on shock wave formation and propagation.

## 2. Methodology

The entire research study integrates three key elements: theoretical foundations, experimental measurements using carefully selected sensors and their application, and CFD analyses utilizing the Ansys Fluent system (Canonsburg, PA, USA), which employs the finite volume method within the framework of continuum mechanics.

In the first step, a combination of experimental measurement and CFD simulation was performed for a pressure gradient between chambers V1 and V2, i.e., *p*1 = 95,000 Pa to *p*2 = 7300 Pa. Pressure measurements were collected at three points on the nozzle wall using pressure sensors in this experimental chamber. Simultaneously, a 2D model for the CFD simulations was developed and validated to ensure its accuracy in representing the specific physical problem. This validated model can now be universally applied to the subsequent analyses presented in this paper.

This paper integrates theory, experiments, and primarily CFD analysis to provide a foundation for planned experiments and theoretically processes the expected results. These analyses are based on, and run parallel to, the theory of the given problem, primarily the theory of one-dimensional isentropic flow. The CFD analysis results facilitate the optimal configuration of the planned experiment, encompassing both design considerations and the selection of suitable experimental conditions, such as pressure ratios and appropriate laboratory conditions for sensor placement. The initial nozzle dimensions were designed based on one-dimensional isentropic flow theory.

As previously noted, the ESEM’s distinctive configuration involves chambers separated by a small aperture subjected to significant pressure gradients, inducing critical flow conditions. A pressure differential drives the gas flow from the higher-pressure chamber toward the lower-pressure chamber, accelerating the gas within the aperture. The flow velocity in the aperture is directly proportional to the increasing pressure difference across it, up to a critical point where the flow velocity reaches 1 Mach [19]. This ratio holds until the aperture flow velocity reaches 1 Mach, marking the onset of critical flow. At this point, the flow velocity is capped at 1 Mach, irrespective of further pressure increases. Consequently, the mass flow rate through the aperture reaches its maximum when the gas velocity attains sonic speed [20,21].

A supersonic flow regime develops downstream of the aperture, characterized by a region of reduced pressure. This phenomenon arises due to the sonic velocity limitation imposed on the gas flow through the aperture [22,23]. The supersonic flow culminates in a shock wave, marked by a significant increase in the gas density [24,25]. The aperture flow is governed by state variables such as the pressure, temperature, density, velocity, and Mach number, which are interconnected according to the principles of one-dimensional isentropic flow. These relationships are detailed in Equations (1)–(6) [26].

(1) $$\frac{v_{v}}{v_{kr}}={\left[\frac{\left(\varkappa +1\right)M^{2}}{2+\left(\varkappa -1\right)M^{2}}\right]}^{\frac{1}{2}}$$

(2) $$\frac{a_{v}}{a_{o}}={\left[\frac{2}{2+\left(\varkappa -1\right)M^{2}}\right]}^{\frac{1}{2}}$$

(3) $$\frac{T_{v}}{T_{o}}=\frac{2}{2+\left(\varkappa -1\right)M^{2}}$$

(4) $$\frac{p_{v}}{p_{o}}={\left[\frac{2}{2+\left(\varkappa -1\right)M^{2}}\right]}^{\frac{\varkappa }{\varkappa -1}}$$

(5) $$\frac{\rho_{v}}{\rho_{o}}={\left[\frac{2}{2+\left(\varkappa -1\right)M^{2}}\right]}^{\frac{1}{\varkappa -1}}$$

(6) $$\frac{\rho_{v}}{\rho_{kr}}=\frac{A_{kr}}{A}=M{\left[\frac{\varkappa +1}{2+\left(\varkappa -1\right)M^{2}}\right]}^{\frac{1}{2}\frac{\varkappa +1}{\varkappa -1}}$$

where *p*_0_ is the input pressure, *p_v_* is the output pressure, *T*_0_ is the input temperature, *T_v_* is the output temperature, *a*_0_ is the input speed of sound, *a_v_* is the output speed of sound, *v_v_* is the output velocity, *v_kr_* is the critical velocity, *ρ*_0_ is the input density, *ρ_v_* is the output density, *M* is the Mach number, ϰ is the gas constant = 1.4, *A* is the computational cross-section, and *A_kr_* is the critical cross-section.

*a*_0_ is the speed of sound in a given medium in the chamber in front of the aperture, where the velocity is null. It is derived from Equation (7):

(7) $$a_{0}=\sqrt{\varkappa RT}$$

where the gas constant for nitrogen ϰ is 1.4, *R* is the universal gas constant and is equal to 287, and *T* is the temperature in a given medium (temperature in chamber V1 is 24 °C).

The fluid used for both the experiments and the CFD simulation was nitrogen, which was treated as a real gas with properties obtained from NIST, not the ideal gas.

The presented relationships (Equations (1)–(6)) served as the foundation for the preliminary design of the nozzle dimensions [27]. The design was based on the investigated pressure ratios and the selected aperture diameter. The specific nozzle dimensions are detailed in the descriptions of the respective solutions. Subsequently, the behavior of the flow, both within this nozzle and particularly downstream of its outlet, was analyzed using the tuned CFD model.

The theory of one-dimensional isentropic flow predicts significant pressure and temperature gradients [28,29]. A density-based solver was employed for the analysis due to its suitability for the given flow conditions. The density-based solver simultaneously solves the governing equations for the continuity, momentum, energy, and species transport, while treating the other scalar equations sequentially. The complex flow dynamics within the nozzle necessitated an implicit linearization approach for solving the coupled equations. The pooled implicit approach, which solves for all the variables simultaneously across the cell faces, demonstrated stability and robustness in handling the complex supersonic flow and steep pressure gradients within the experimental chamber.

Given the nature of supersonic flow, viscous dissipation effects were incorporated into the energy equation by activating the corresponding terms. Viscous heating becomes significant as the Brinkman number (*B_r_*) approaches unity [30]. Owing to the extremely high flow velocity within the nozzle, the heat transfer is negligible.

(8) $$B_{r}=\frac{\mu U_{e}^{2}}{k\Delta T}$$

where ΔT is the temperature difference in the system.

Given the high flow velocities and the negligible heat transfer between the flow and the wall, adiabatic conditions were assumed for the walls.

Next, to discretize the convective and compressive flow terms, the advection upstream splitting method (AUSM) was employed. This scheme leverages the eigenvalues of the Jacobian flow matrices.

The AUSM scheme offers several advantages, including the following:

- Accurate capture of shock and contact discontinuities.
- Entropy-conserving solution capabilities.
- Suppression of the carbuncle phenomenon, a common numerical instability associated with low-dissipative shock-capturing schemes.
- Robust accuracy and convergence across a wide Mach number range.

Significantly, the method’s effectiveness does not rely on explicit eigenvector information, making it suitable for systems with complex, undefined eigenstructures, such as those encountered in two-fluid multiphase flow models [30,31].

A second-order upwind scheme, utilizing multivariate linear reconstruction, was employed for inter-cell data transfer [32]. By employing the Taylor series expansion of the cell-centered solution around the cell centroid, higher-order accuracy is achieved at the cell faces [33,34].

The present approach accurately captured the dynamic flow variations during pumping, aligning well with the experimental data. The rigorous CFD modeling necessitated a meticulously refined mesh [35].

**Figure 2:**
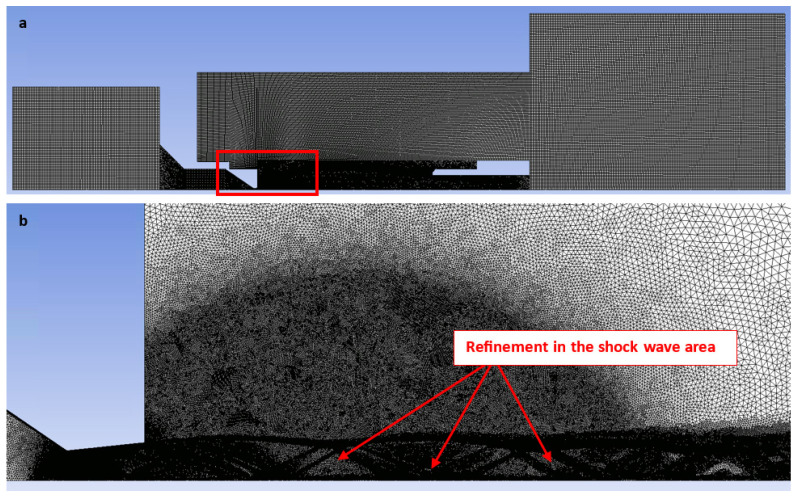
Structured mesh for the CFD analysis (**a**), with the zoomed area showing the mesh refinement (**b**).

A structured mesh combining 2D structured rectangular elements with unstructured triangular elements was implemented. This approach mitigated the numerical artifacts associated with oblique interfaces and optimized the cell count in predominantly rectangular regions (Figure 2a). Triangular elements were strategically employed in regions where a structured mesh could not be generated, particularly within the narrow nozzle aperture and anticipated supersonic flow zones characterized by significant pressure and density gradients. A sufficiently fine boundary layer was modeled within this aperture and nozzle [36,37]. Figure 2b illustrates an expanded rectangular domain (shown in Figure 2a), with a zoomed inset highlighting the refined mesh region. Additionally, manual adaptive refinement based on pressure gradient criteria was applied during the calculation process using the field variable method. The refinement was focused on regions exhibiting oblique and normal shock waves [38].

The mesh adaptation was driven by the maximum pressure gradient values, employing a cell-based derivative approach and a maximum refinement level of 4. This effectively captured the pressure gradients within the supersonic nozzle flow.

Next, a grid independence study was conducted, employing manual mesh adaptation across the pressure range. A cell-in-range approach with a maximum refinement level of 2 was utilized in regions of minimal variable change. Conversely, a maximum refinement level of 4 was applied to areas upstream of the aperture, within the nozzle, and in the gas expansion zone.

The initial cell size at the wall, particularly within the boundary layer at the aperture and nozzle, was crucial for the mesh generation. The SST k-ω turbulence model was employed, assuming a *y*^+^ value within the range of 0 and 1.

The size of the first cell adjacent to the wall is obtained from Equation (9):

(9) $$y=\frac{y^{+}\mu }{U_{\tau }\rho }$$

where *y^+^* is a dimensionless quantity that represents the distance from the wall within the boundary layer, scaled according to the flow characteristics, *μ* is the dynamic viscosity, and *U_τ_* is the frictional velocity.

Given the employed k-ω turbulence model, which does not incorporate a mathematical wall function model, the value must be less than 1. To ensure accurate representation of the boundary layer, the mesh must be sufficiently refined in this region, and the first cell adjacent to the wall must be small.

The dynamic viscosity, dependent on the gas temperature *T* and exhibiting significant variation in supersonic flow, is obtained from Equation (10) [39] and verified according to [40]:

(10) $$\mu =\frac{{1.38421.T}^{1.5}}{\left(T+103.874\right)}$$

The frictional velocity *U_τ_* is determined from Equation (11):

(11) $$U_{\tau }=\sqrt{\frac{\tau_{w}}{\rho }}$$

where $\tau_{w}$ is the wall shear stress determined from Equation (12):

(12) $$\tau_{w}=\frac{1}{2}C_{f}\rho U_{max}^{2}$$

where *U_max_* is the maximal gas flow velocity in the flow axis, and *C_f_* is the skin friction coefficient.

The skin friction coefficient is a dimensionless quantity that quantifies the ratio of the frictional force acting on the surface of a body to the dynamic pressure of the flowing fluid. It is determined using Equation (13):

(13) $$C_{f}=0.058{Re}_{l}^{-0.2}$$

where ${Re}_{l}$ is the Reynold number obtained from Equation (14):

(14) $${Re}_{l}=\frac{\varrho U_{mid}D}{\mu }$$

where *D* is the characteristic dimension of the internal solved space, and *U_mid_* is the mean flow velocity.

The mean flow velocity *U_mid_* in the cross-section of the given nozzle is determined from Equation (15):

(15) $$U_{mid}=\frac{U_{max}}{2}$$

These relationships were subsequently utilized in the evaluation of the individual calculation variants.

The Ansys Fluent monitoring setup remained unchanged throughout the subsequent calculation stages. Global parameters, including the absolute pressure, static temperature, velocity, and density, were continuously monitored. Specific parameter points were defined within the aperture throat and at five locations spaced 2 mm apart along the post-aperture gas flow axis. Similarly, the subsequent evaluations of the relevant quantities, as detailed later, demonstrated consistent behavior. A grid independence analysis confirmed the adequacy of the mesh resolution for the given analysis.

Since this paper involves varying pressures, which significantly alter the ratio of inertial and viscous forces, as mentioned previously, the Reynolds number *Re* was evaluated. The Reynolds number quantifies the relative importance of inertial forces to viscous forces (i.e., resistance due to internal friction). This is crucial in our analysis, as low-pressure conditions result in a different ratio of inertial to viscous forces compared to atmospheric pressure. Furthermore, the Reynolds number determines whether the flow is laminar or turbulent. Laminar flow occurs at low Reynolds numbers, characterized by smooth, orderly fluid particle movement along parallel paths. Turbulent flow, conversely, occurs at high Reynolds numbers and is characterized by chaotic fluid particle motion, vortices, and turbulence. The typical threshold values for distinguishing between laminar and turbulent flow are commonly reported as the following:

Re < 2300    laminar flow

2300 < Re < 4000 transient flow

Re > 4000    turbulent flow

The Reynolds number is calculated according to the following Equation (16):

(16) $$Re=\frac{\rho \cdot v\cdot d}{\eta }$$

where *Re* is the Reynolds number, ρ is the fluid density, *v* is the flow velocity, *d* is the characteristic dimension, and *η* is the dynamic fluid viscosity.

Given the high velocities and significant temperature drops encountered in this paper, it was necessary to incorporate Sutherland’s law (Equation (17)). Sutherland’s law describes the temperature-dependent behavior of dynamic viscosity:

(17) $$\eta =\eta_{0}\cdot \left(\frac{a}{b}\right)\cdot {\left[\frac{T}{T_{0}}\right]}^{\frac{3}{2}}$$

where $\eta_{0}$ is the reference dynamic viscosity at reference temperature *T*_0_, *T* is the input temperature, *T*_0_ is the reference temperature, *a* is 0.555*T*_0_ + *C*, *b* is 0.555*T* + *C*, and *C* is Sutherland’s constant.

**Figure 3:**
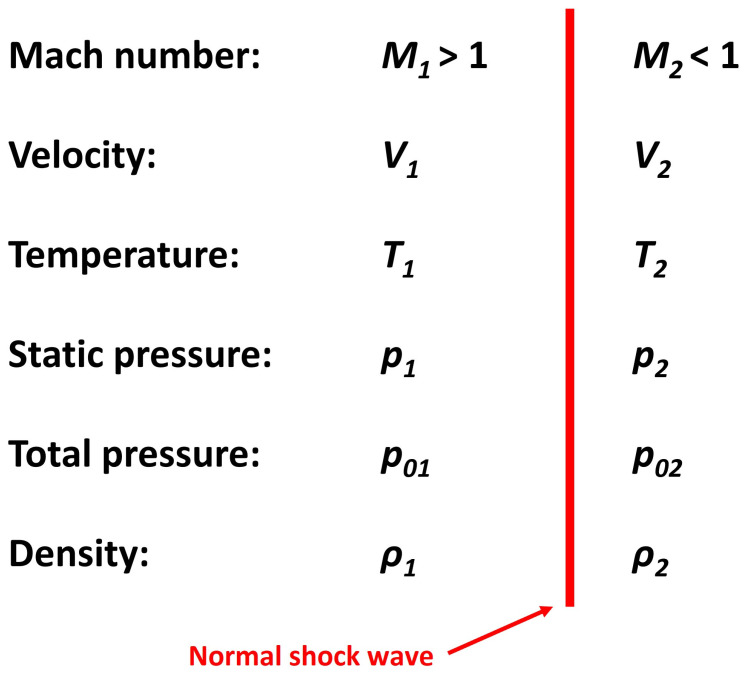
Changes in the state variables across a normal shock wave.

Finally, the CFD analysis results were validated against the second part of the one-dimensional isentropic flow theory (Equations (18)–(24)) [26], which addresses changes in the state variables across a normal shock wave (Figure 3). The primary objective of this comparison was to validate the results obtained from the CFD analyses.

(18) $$M_{1n}=M_{1}\sin\alpha_{s}$$

(19) $$M_{2}^{2}=\frac{2+\left(\varkappa -1\right)M_{1n}^{2}}{2\varkappa M_{1n}^{2}-\left(\varkappa -1\right)}$$

(20) $$\frac{T_{2}}{T_{1}}=1+\frac{2\left(\varkappa -1\right)}{{\left(\varkappa +1\right)}^{2}}\cdot \frac{1+\varkappa M_{1n}^{2}}{M_{1n}^{2}}\cdot \left(M_{1n}^{2}-1\right)$$

(21) $$\frac{\rho_{2}}{\rho_{1}}=\frac{V_{1}}{V_{2}}=\frac{\left(\varkappa +1\right)M_{1n}^{2}}{2+\left(\varkappa -1\right)M_{1n}^{2}}$$

(22) $$\frac{p_{02}}{p_{01}}={\left[1+\frac{2\varkappa }{\varkappa +1}\left(M_{1n}^{2}-1\right)\right]}^{-\frac{1}{\varkappa -1}}{\left[\frac{\left(\varkappa +1\right)M_{1n}^{2}}{2+\left(\varkappa -1\right)M_{1n}^{2}}\right]}^{\frac{\varkappa }{\varkappa -1}}$$

(23) $$\frac{p_{02}}{p_{1}}={\left[1+\frac{2\varkappa }{\varkappa +1}\left(M_{1n}^{2}-1\right)\right]}^{-\frac{1}{\varkappa -1}}{\left[\frac{\varkappa +1}{2}M_{1n}^{2}\right]}^{\frac{\varkappa }{\varkappa -1}}$$

(24) $$\frac{p_{2}}{p_{1}}=1+\frac{2\varkappa }{\varkappa +1}\left(M_{1n}^{2}-1\right)$$

where *M*_1n_ is the normal component of the Mach number, *M*_2_ is the Mach number behind the normal shock wave, *T*_2_ is the temperature behind the shock wave, *T*_1_ is the temperature in front of the shock wave, *p*_2_ is the static pressure behind the shock wave, *p*_1_ is the static pressure in front of the shock wave, *ρ*_2_ is the density behind the shock wave, *ρ*_1_ is the density in front of the shock wave, *p*_02_ is the total pressure behind the shock wave, and *p*_01_ is the total pressure in front of the shock wave.

The results obtained from the experimental measurement and CFD simulation were compared with those obtained using the optical method. A single spherical mirror system was employed for the shock wave imaging, which exhibits significantly higher sensitivity to changes in the medium’s density than the Toepler system due to the double passage of photons through the inhomogeneity (supersonic gas flow).

**Figure 4:**
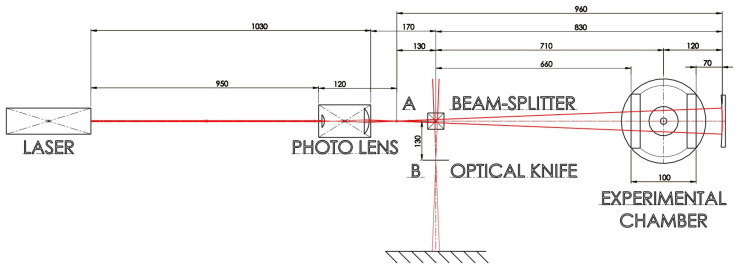
Used optical system.

The optical system utilized a helium–neon laser HRR015 with a power of up to 2 mW, emitting a collimated beam with a diameter of 0.57 mm and a wavelength of 632.8 nm. The collimated laser beam passes through a Canon EF 28–80 mm f/3.5–5.6 V USM photo lens (Tokio, Japan), focused so that the resulting main focus A of the scattered beam is 120 mm behind the first of the objective lens array. Further along the path of the laser beam is a 25 mm beam-splitter, which, while deflecting 50% of the beam away before the experimental chamber, allows the resulting image of the supersonic jet in the chamber to be easily captured. This beam-splitter is located 130 mm behind the main focal point A of the photo lens in order to obtain a secondary focal point B outside the original optical axis, respectively, 130 mm away from it. Behind the beam-splitter is an experimental chamber in the beam axis, the center of which is 710 mm from the beam-splitter. Moreover, 120 mm behind the center of the chamber, which also means 830 mm behind the beam-splitter or 960 mm from the main focal point, is a spherical mirror with a radius of curvature of 480 mm. The mirror is mounted on a tunable fixture for easy control of its tilt and is positioned and adjusted so that the focal point of the laser beam reflected from it (secondary focal point B) overlaps with the original main focal point A of the beam coming out of the photo lens when the beam-splitter is removed. This eliminates the image doubling that would otherwise occur due to different photon paths to and from the mirror. The laser beam travelling back from the mirror through the inhomogeneity, carrying information about changes in the density of the medium along its path, is reflected from the beam-splitter to the opposite side to the first diverted half of the original beam. Due to the correct mirror alignment, the secondary focal point B is located on the diverted path 130 mm from the center of the beam-splitter, where an optical knife (aperture) is placed to cut off the part of the beams diverted from the original trajectory by the investigated inhomogeneity. Behind the optical knife, the trimmed laser beam is incident on a screen, where it is captured by a camera (Figure 4).

To establish a scale for the photographs, 0.5 mm diameter wires were placed on the outer walls of the experimental chamber visors. The shadow widths of the individual wires were measured in pixels on the acquired photographs and then averaged to determine a scale for the inhomogeneity image located in the middle of the experimental chamber, i.e., at a distance midway between the calibration wires.

## 3. Results

### 3.1. Comparison of Experiment and CFD Simulation

**Figure 5:**
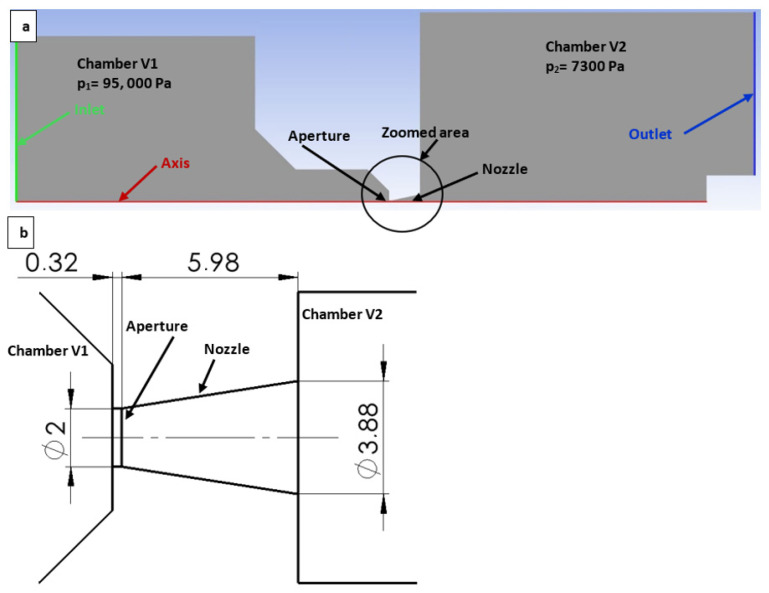
Two-dimensional axisymmetric model of the chambers for the CFD analysis, with labeled boundary conditions (**a**) and with the zoomed area showing its dimensions (mm) (**b**).

Initially, CFD analyses were conducted on a 2D axisymmetric model within the Ansys Fluent system. The boundary conditions for this simulation are illustrated in Figure 5a. The nozzle dimensions were carefully selected to investigate the flow separation from the boundary layer for a slightly overexpanded nozzle. These dimensions were determined based on the theoretical considerations for the computational cross-section (Figure 5b).

From Equation (6), the computational cross-section area was determined for pressure ratios ranging from 7300 Pa to 95,000 Pa, resulting in a ratio of 0.0769. For a critical cross-section area in the aperture of 3.14 mm², the corresponding output cross-section area in the nozzle is 7 mm², leading to a diameter of 3 mm. In our case, the nozzle was designed to be overexpanded to facilitate the analysis of the flow through the nozzle with increased back pressure and the formation of normal shock waves, as well as for other planned experiments.

**Figure 6:**
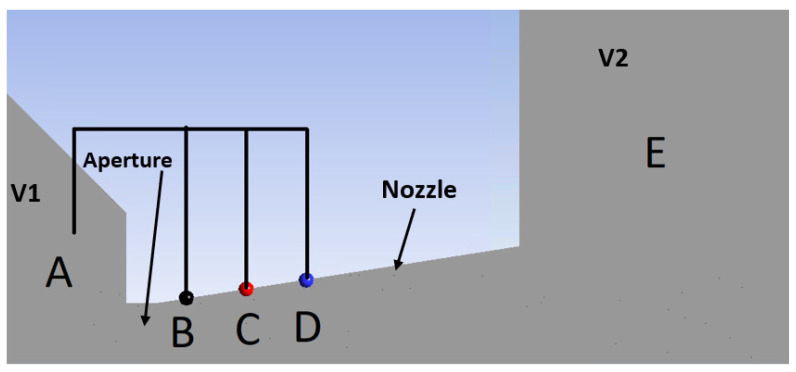
Points of the pressure measurements on the nozzle wall in the experimental chamber.

Subsequently, pressure measurements were conducted at points indicated in Figure 6 on the nozzle wall within the experimental chamber. Point A was measured using an absolute pressure sensor, the Pfeiffer CMR 361, with a range of 110 kPa and an accuracy of ±0.2% of the measured value. Point E was measured using an absolute pressure sensor, the Pfeiffer CMR 362, with a range of 11 kPa and an accuracy of ±0.2% of the measured value. The values of the remaining points, B, C, and D, were obtained using differential sensors:

- The differential pressure between points A and B was measured to be 74,400 Pa using a DPS 300 sensor with a range of 100 kPa and an accuracy of ±1% FSO BFSL (over the entire range fitted with a linear curve).
- The differential pressure between points B and C was measured to be 8500 Pa using a DPS 300 sensor with a range of 25 kPa and an accuracy of ±1% FSO BFSL (over the entire range fitted with a linear curve).
- The differential pressure between points C and D was measured to be 4500 Pa using a DPS 300 sensor with a range of 4 kPa and an accuracy of ±1% FSO BFSL (over the entire range fitted with a linear curve).

The pressure values presented in Table 1 were measured using the described pressure sensors and compared to the results obtained from the CFD simulation. The relative error *ε* is also included in Table 1.

**Figure 7:**
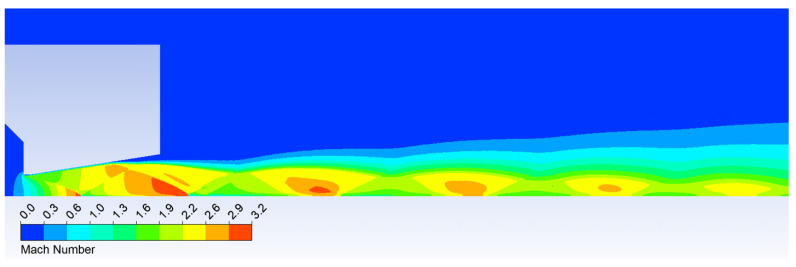
Mach number distribution.

Figure 7 depicts the Mach number distribution, clearly illustrating a constriction of the flow exiting the nozzle due to the aforementioned increased back pressure at the nozzle outlet. At the same time, sharp changes in velocity can already be seen in the regions with a normal shock wave, with a steeper gradient than in the regions with an oblique shock wave.

**Figure 8:**
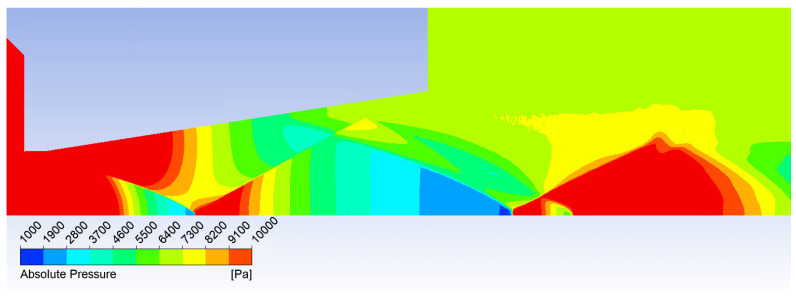
Static pressure distribution.

**Figure 9:**
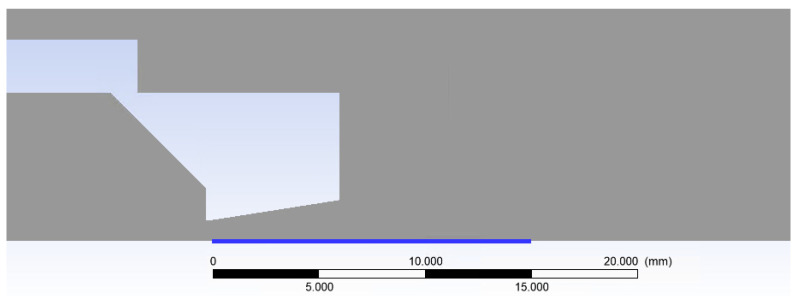
Path along which selected state variables are plotted (blue line).

The Mach number distribution directly influences the static pressure distribution (Figure 8). The distribution of pressure gradients provides insights into the anticipated shock wave pattern. A path along which selected state variables are plotted is illustrated in Figure 9.

**Figure 10:**
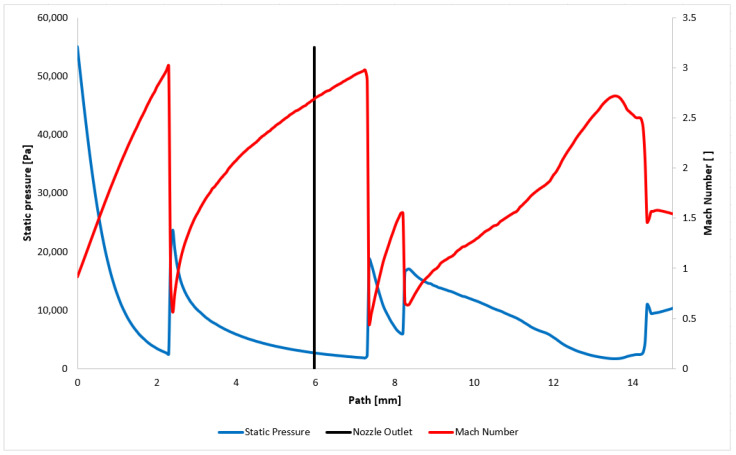
Static pressure and Mach number layout on the path.

The static pressure and Mach number distributions are plotted in Figure 10. These distributions clearly indicate the presence of normal shock waves at positions 2.3 mm, 7.8 mm, and 8.3 mm, where the Mach number drops below 1 and the static pressure increases sharply. The first shock wave occurs within the nozzle itself, rendering it undetectable using the current optical method configuration. The other two are located downstream of the nozzle outlet and are further mapped.

**Figure 11:**
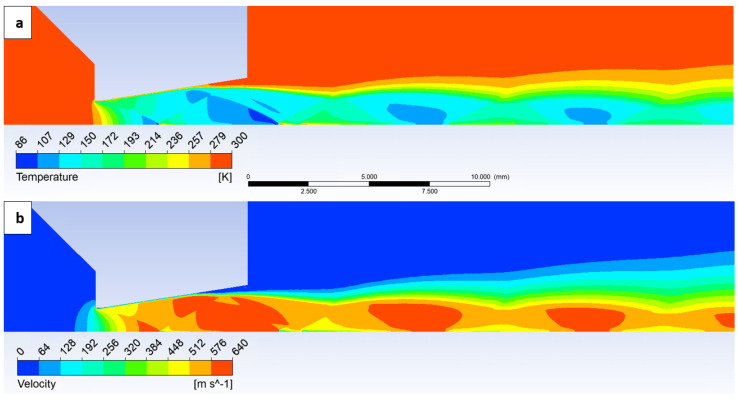
Static temperature (**a**) and velocity (**b**) distribution on the path.

The static temperature distribution was also mapped, which, in conjunction with the Mach number and static pressure, provides a comprehensive description of the supersonic flow characteristics. It is also important for evaluating two facts. As depicted in Figure 11a, the temperature profile, particularly within the boundary layer, exhibits non-uniformity. This deviation is influenced by oblique shock waves and the boundary layer itself. Consequently, for the application of one-dimensional isentropic flow theory, it would be necessary to utilize the average values across the cross-section. The second fact supports the applicability of optical methods at low pressures. The minimum pressure required for successful application of the Schlieren method depends on several factors, including the temperature differences. The greater the temperature differences in the environment, the greater the density variations and the better the visibility in the Schlieren image. The velocity distribution, as presented in Figure 11b, is essential for evaluating the Reynolds number.

**Figure 12:**
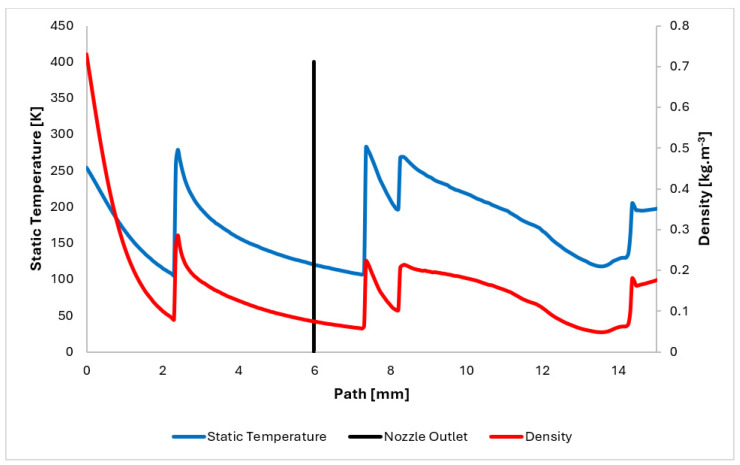
Static temperature and density layout on the path.

The temperature profile along the path plotted in Figure 12 is directly influenced by the pressure, density, and Mach number distributions. It exhibits a decrease into cryogenic temperatures within the supersonic region.

Figure 12 also depicts the density distribution. This distribution reveals significant gradients, comparable in nature to those observed in the pressure gradients. Notably, these large gradients occur at positions 2.3 mm, 7.8 mm, and 8.3 mm.

Optical setups at such low pressures, as investigated in this paper, are highly challenging to configure. This is part of a research study aimed at optimizing these optical setups for operation within the pressure range of differentially pumped chambers. The minimum pressure required for the successful application of Schlieren methods is influenced by several factors, including the type of gas, temperature variations in the environment, the geometry of the optical system, and the sensitivity of the detection system.

**Figure 13:**
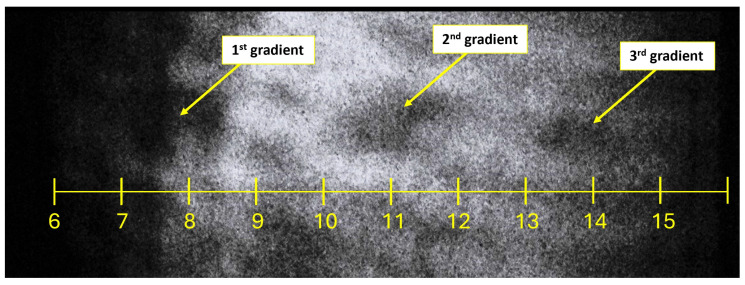
First velocity derivative imaging.

Figure 13 presents the distribution of the first derivative of the velocity, which corresponds to the rate of change of the density. The optical configuration described previously was employed, and the flow was observed through the windows installed in the experimental chamber (Figure 1a). Gradient regions were identified starting at distances of 7.3, 10.75, and 14.2.

The gradient observed at the position of 7.3 mm corresponds to the most significant changes within the region of the normal shock wave visible in Figure 12. The less pronounced region at a distance of 10.75 mm along the path signals a disruption to the density decrease at around 12 mm. The region at 14.2 mm indicates the presence of another normal shock wave.

It is acknowledged that Figure 13 contains noise, including other darker regular spots caused by reflections within the experimental chamber. To mitigate these artifacts, the interior of the chamber will require blackening. The primary focus of this paper was the fine-tuning of the experimental chamber and CFD simulations, which serve as a foundation for the gradual adjustment of the optical apparatus, which has been accomplished.

**Figure 14:**
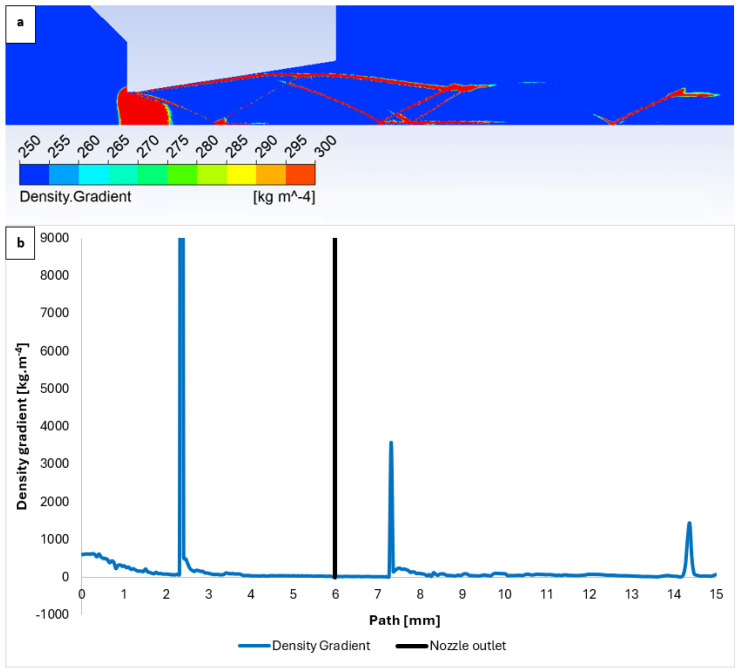
Graphical density gradient distribution (**a**) and density gradient layout on the path (**b**).

Figure 14a presents the distribution of the density gradient, as evaluated using an optical method. The gradients at the previously mentioned locations, along with their intensity, are clearly visible in Figure 14b.

**Figure 15:**
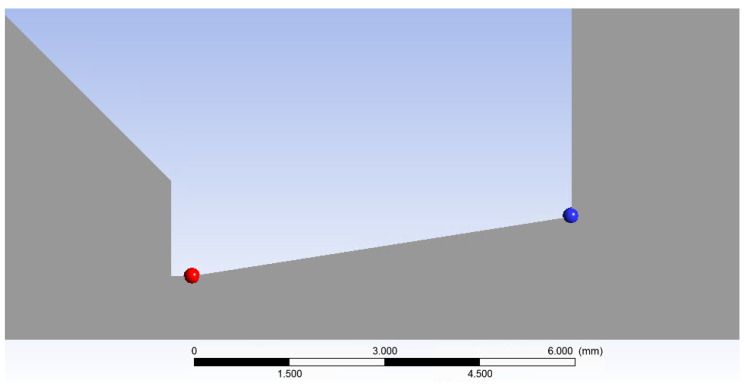
Locations for the *y+* verification.

The *y+* value was verified according to the theory described in the Methodology. The required maximum size of the first cell at the wall was determined at two locations: the aperture edge and the nozzle outlet (Figure 15). Table 2 shows the results of the first cell size *y* at the given points.

**Figure 16:**
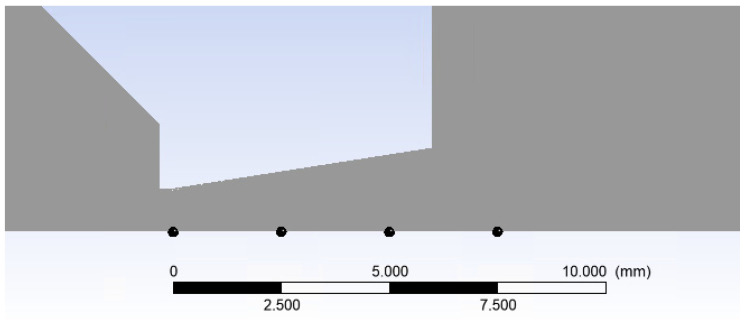
Points for the Reynolds number verification.

Additionally, the Reynolds number was calculated to determine the flow characteristics in the region where inertial forces and viscous forces interact. Four points were chosen (Figure 16), from which the reference values of the state variables were taken. The results of this evaluation are presented in Table 3.

### 3.2. CFD Analyses of the Influence of the Pressure Magnitude on the Shock Wave Location

The previous results obtained with the existing nozzle in the experimental chamber, primarily intended for the study of the nozzle flow, have demonstrated, among other things, that shock waves form within the nozzle for the expected range of pressure ratios. To facilitate optical measurement studies, it is advisable to design a new nozzle for the interchangeable adapter, specifically a shorter nozzle. To achieve these goals, the following CFD analyses were performed to prepare the groundwork for further fine-tuning of the optical system and experimental measurements for a wider range of pressures, from atmospheric to the low pressures employed in the ESEM. The research aims to investigate the differences in the characteristics of supersonic flow at low pressures, which occur during the pumping of vacuum chambers in the ESEM under operational conditions.

**Figure 17:**
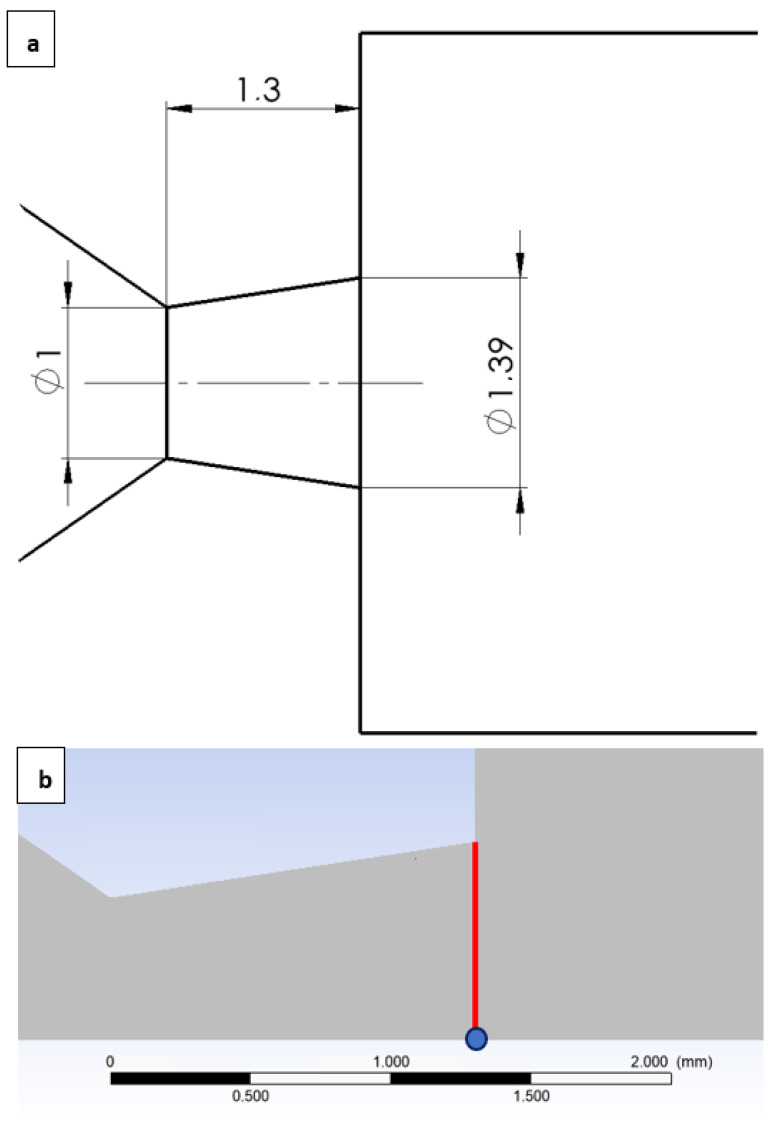
Design of the shortened nozzle with its dimensions (mm) (**a**) with an emphasized nozzle outlet diameter (line) with the point for the values from the CFD simulations (**b**).

For this reason, a shortened nozzle shape was designed in the calculated shape. The presented equations (Equations (1)–(6)) were employed to determine the dimensions of this nozzle. The design was based on the investigated pressure ratio *p_v_*:*p*_0_ = 0.1 and the selected aperture diameter of 1 mm. The pressure range within chamber V2 was varied from atmospheric pressure down to 2000 Pa. The specific pressure ratios and their corresponding designations are tabulated in Table 4. According to Equation (4), for the aforementioned pressure ratio, the Mach number at the nozzle outlet would be 2.16. Subsequently, the remaining values, as calculated from the other relations, are presented in Table 5. These calculations resulted in a nozzle outlet diameter of 1.39 mm (Figure 17a).

The results were compared against the one-dimensional isentropic flow theory as outlined by the other presented relationships (Equations (1)–(6)). The results are summarized in Table 6, where the column POINT presents the values obtained from the CFD analyses at the nozzle outlet along the flow axis, and the column LINE shows the average value of the given quantity across the entire cross-section (Figure 17b). This comparison was conducted because the isentropic one-dimensional flow theory assumes an ideal gas with no internal friction and neglects the boundary layer effects, thus assuming that the values of the state variables are uniform across the nozzle cross-section.

In this paper, nitrogen was considered a real gas with properties obtained from NIST, rather than an ideal gas, accounting for the internal friction and boundary layer effects. Consequently, measurements taken at a single point do not accurately represent the true behavior of the flow. However, the average values exhibit reasonable agreement, and the slight discrepancy can be attributed to the differences in the real gas properties used in the CFD simulations.

**Figure 18:**
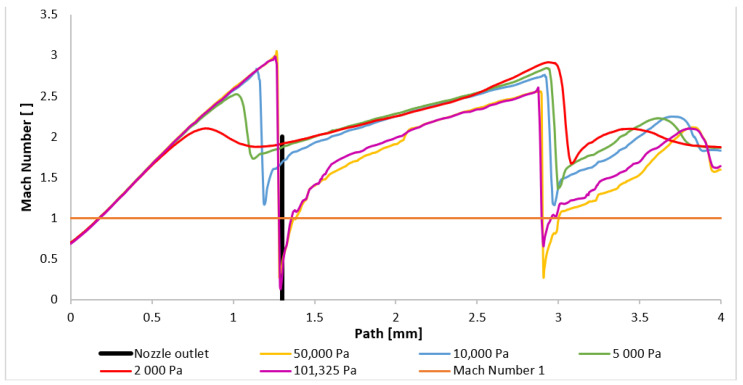
Mach number layout of the shortened nozzle for each variant on the path (axis).

The results also confirmed the anticipated finding that, as the gas exits from the nozzle, normal shock waves do not develop near the computational cross-section, contrary to the findings in [21]. Instead, oblique shock waves intersect. Figure 18 illustrates this, demonstrating that the Mach number remains almost consistently above 1 Mach, with only a few exceptions in cases with higher pressures. The observed deviations can be attributed to the non-ideal, real-world nature of the flow, as opposed to an idealized isentropic one-dimensional flow.

This posed a challenge for our intended research, which was predicated on the analysis of normal shock waves. To address this, the nozzle outlet diameter was reduced to 1.273 mm, resulting in an underexpanded nozzle configuration.

**Figure 19:**
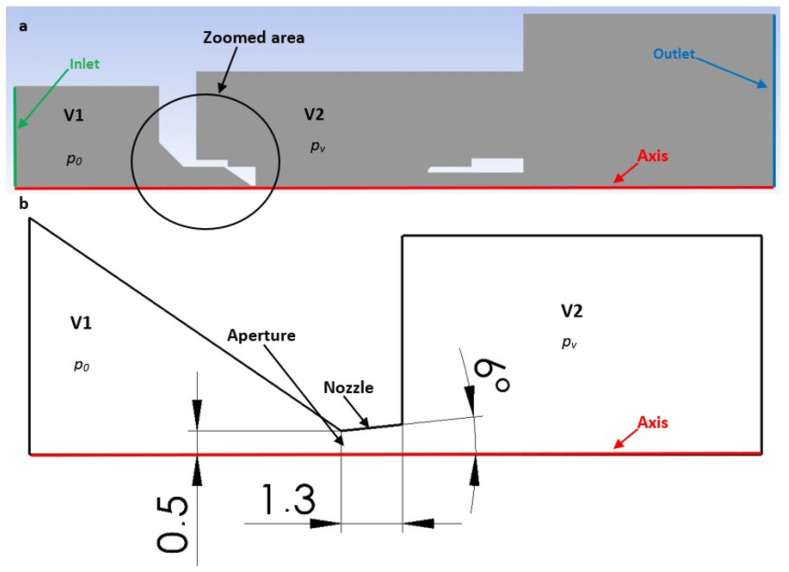
Two-dimensional axisymmetric model of the underexpanded nozzle of the chambers for the CFD analysis with labeled boundary conditions (**a**) and with the zoomed area showing its dimensions (mm) (**b**).

Figure 19a presents a 2D axisymmetric model of the underexpanded nozzle, as used for the CFD analysis, along with a description of the boundary conditions. The chamber behind the aperture is denoted as V1, and the chamber into which the gas flows through the aperture and nozzle is denoted as V2. Figure 19b illustrates a zoomed-in region showcasing the location of the aperture, nozzle, and model dimensions.

**Figure 20:**
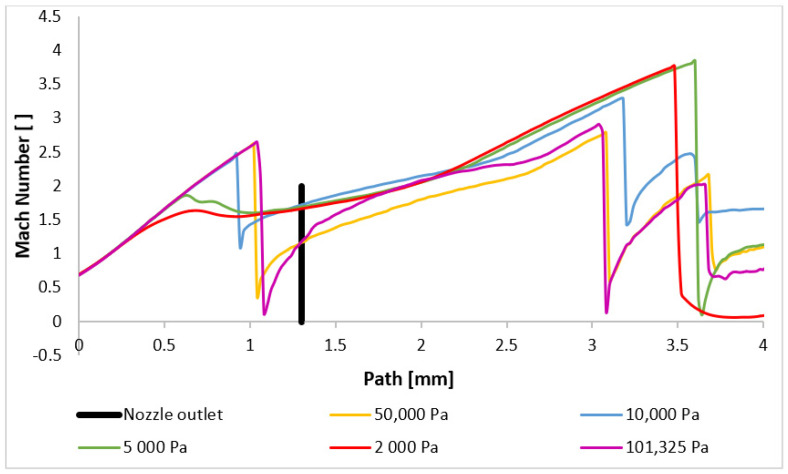
Mach number layout of each variant on the path (axis).

**Figure 21:**
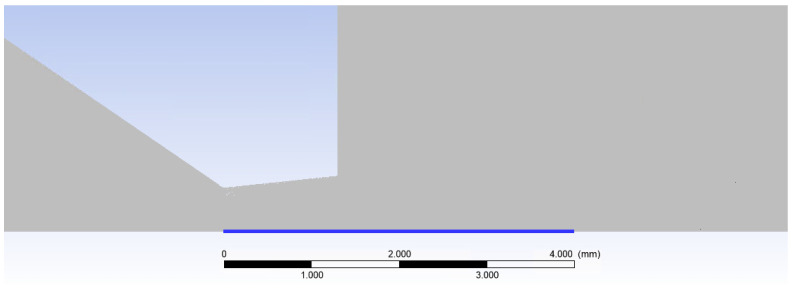
Axis of the flow (blue line—path).

**Figure A1:**
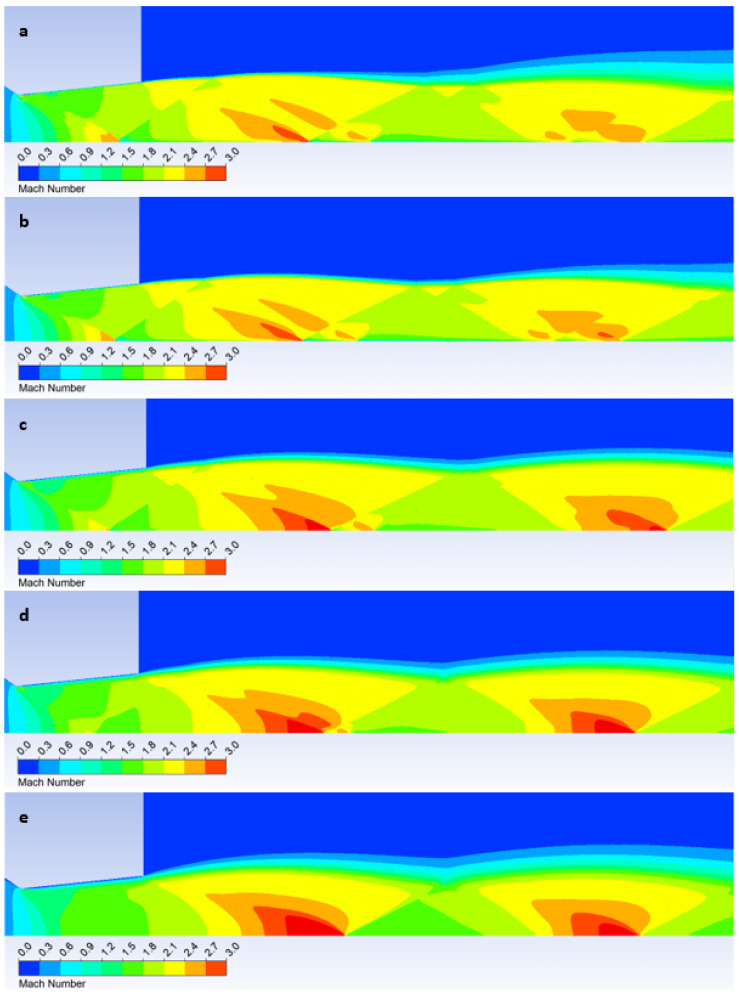
Mach number distribution of each variant: 101,325 Pa (**a**), 50,000 Pa (**b**), 10,000 Pa (**c**), 5000 Pa (**d**), and 2000 Pa (**e**).

The character and formation of shock waves are significantly influenced by the supersonic flow pattern, which is quantified by the Mach number. The initial analysis involves evaluating the Mach number distribution. Figure 20 presents a comparison of the overall distribution of this parameter in the axial direction (path) at a distance of 4 mm from the aperture (Figure 21). The investigated region extends from the narrowest cross-section of the nozzle, represented by the aperture located at point zero, to the end part of the nozzle, where the first and second shock waves form at higher pressures. Behind it, at elevated pressures, the formation of a third and additional shock waves is observed, as depicted in Appendix A (Figure A1a–e).

Figure 20 illustrates that for pressures ranging between 101,325 Pa and 10,000 Pa, the flow velocity within the nozzle decreases to subsonic speeds at a distance between 0.9 mm and 1.1 mm before accelerating back to supersonic speeds. However, for pressures below 10,000 Pa, the flow velocity remains supersonic throughout the examined region. Their profiles, in our case variants 5000 and 2000 Pa, exhibit significant differences. These variations directly influence the subsequent development of supersonic flow, as discussed below.

**Figure 22:**
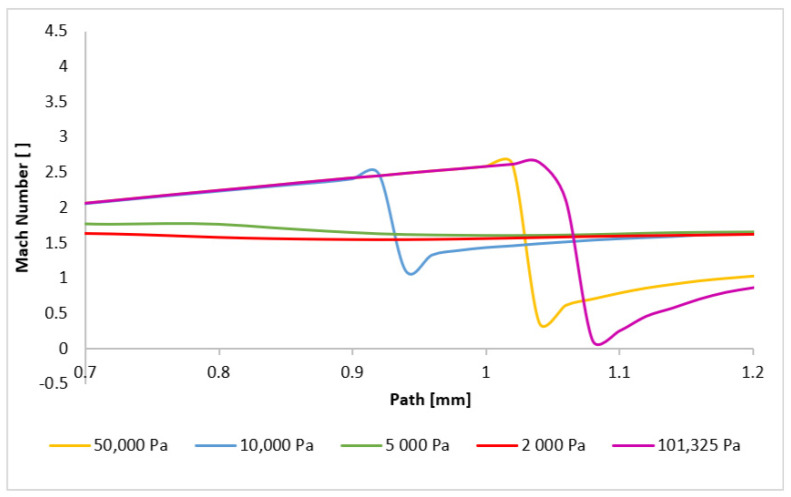
Mach number layout of each variant on the path (axis) with the adjusted scale.

It is noteworthy that the transition to supersonic flow, characterized by the formation of a normal shock wave, occurs at varying distances from the aperture for all the variants, directly related to the pressure value. The formation of the first normal shock wave within the nozzle occurs at its furthest distance for the variant with the highest pressure, namely 101,325 Pa. Conversely, as the pressure in chamber V2 decreases, the formation of the normal shock wave moves closer to the aperture. This phenomenon is more evident in Figure 22, where the scale has been adjusted to magnify the region of the initial drop in the flow velocity below 1 Mach. For pressures below 10,000 Pa, a significant change in behavior is observed. While for the entire pressure range from 101,325 Pa to 10,000 Pa, the velocity profile remains similar, only the distance of the first normal shock wave formation from the aperture decreases. Additionally, the magnitude of the velocity drop below 1 Mach decreases. However, for pressures below 10,000 Pa, the velocity profile is entirely different. There is a slight decrease in the flow velocity, but it does not drop below 1 Mach. Thus, a normal shock wave is not formed, but a slight decrease in velocity for the 2000 Pa and 5000 Pa variants is caused by an oblique shock wave. The reason for the lower velocity drop in the variants with lower pressure lies in the differing ratio of inertial and viscous forces. At lower pressures, the influence of viscous forces becomes more pronounced, hindering the formation of a thinner and more intense normal shock wave by the lower inertial forces. This phenomenon was previously discussed in the Methodology section. The reduced velocity drop subsequently affects the location and characteristics of the second normal shock wave.

**Figure 23:**
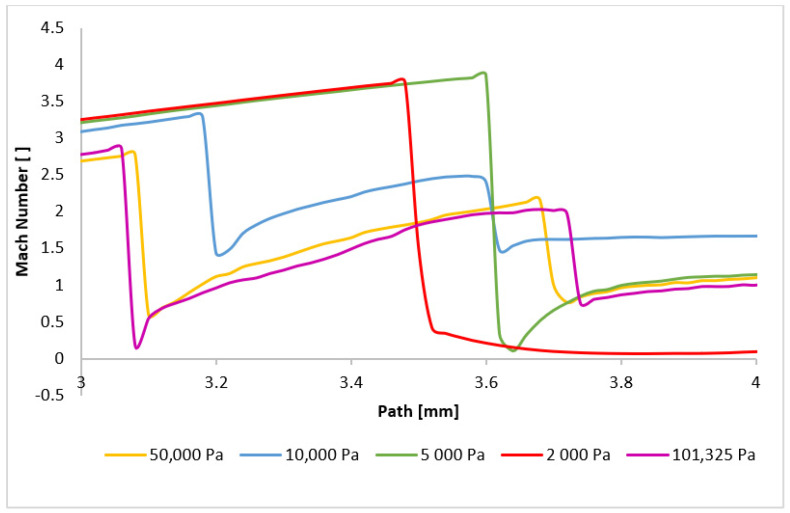
Mach number layout of each variant on the path (axis) with another adjusted scale.

The presented results directly influence the phenomena occurring within the examined path, particularly in the region between 3 mm and 3.6 mm where aperture-dependent effects are observed. Figure 23 provides a magnified view of this region, revealing that for the pressure variants exhibiting subsonic velocities in the aperture, a subsequent velocity drop below 1 Mach occurs after a second normal shock wave. Interestingly, the order of shock wave formation is reversed for these cases. Higher pressures result in shock waves closer to the aperture. For example, the 101,325 Pa variant exhibits the closest shock wave formation, while the 50,000 Pa variant forms it further away. This behavior can be attributed to the reduced velocity drop across the initial shock wave in the nozzle for lower pressures. Consequently, the subsequent velocity increase is less pronounced, delaying the formation of the second shock wave. Periodically, a third normal shock wave appears at approximately 3.7 mm. The changes in flow characteristics are even more pronounced for the boundary case of 10,000 Pa and the lower pressure variants. Due to the marginal drop below 1 Mach at the first normal shock wave, the boundary variant experiences the most rapid velocity increase after this first shock wave, and the subsequent decrease occurs at a distance of almost 3.2 mm, followed by pulsations above 1 Mach. For the 5000 Pa and 2000 Pa variants, where the flow velocity does not drop to subsonic speeds in the aperture, the pulsations and velocity decreases are caused solely by oblique shock waves, without the abrupt changes induced by normal shock waves. This results in a deceleration of the flow to subsonic speeds at a significant distance for both variants, as can be seen in Appendix A (Figure A1a–e).

**Figure 24:**
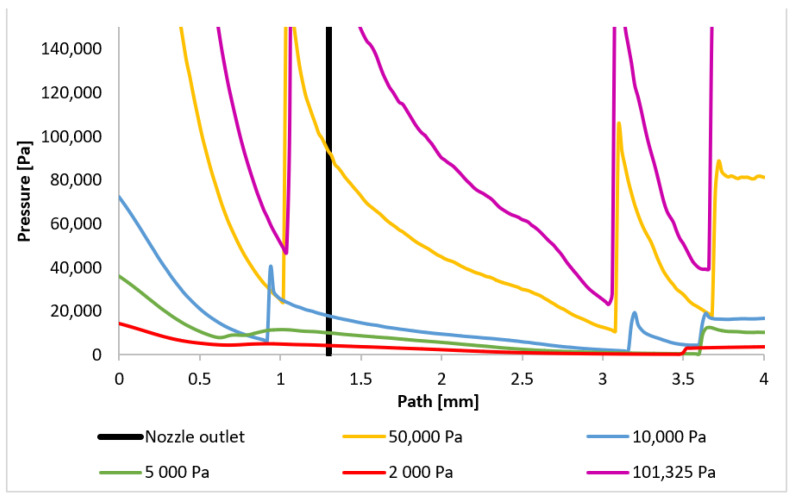
Pressure layout of each variant on the path (axis).

The Mach number distribution directly influences the pressure profile, which is strongly dependent on the flow velocity. Figure 24 presents the pressure distribution along the path (Figure 21), clearly indicating the presence of pressure gradients at locations corresponding to normal shock waves. Three distinct regions of shock wave formation are evident: around 1 mm, approximately 3.1 mm, and at 3.6 mm along the path.

**Figure 25:**
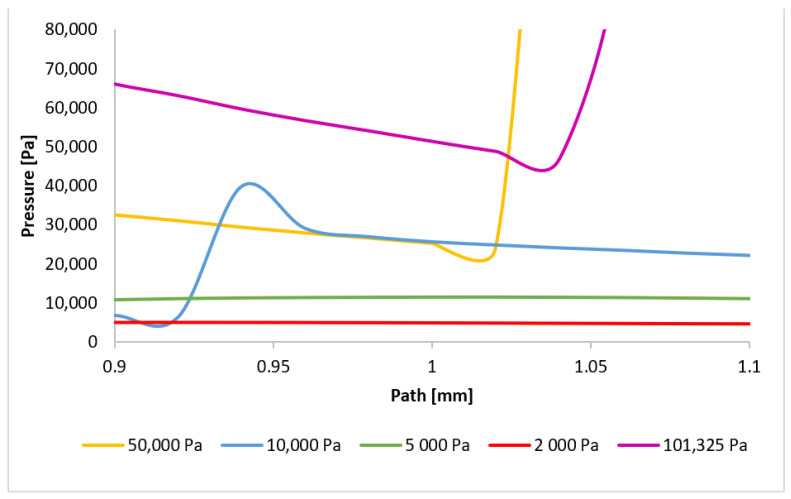
Pressure layout of each variant on the path (axis) with the adjusted scale.

The phenomena occurring in the initial region will be examined first. As previously discussed, for pressures below 10,000 Pa, the region around 1 mm (within the nozzle) does not exhibit pressure gradients associated with normal shock waves. However, for higher pressures, the decrease in velocity below 1 Mach induces significant pressure gradients, directly related to the subsequent velocity increase and eventual drop to subsonic speeds. Consequently, the pressure gradient for the 101,325 Pa variant is located furthest along the path, with subsequent gradients occurring closer to the aperture. These locations will be precisely determined by analyzing the plotted pressure gradients. Figure 25 provides a magnified view of the region around 1 mm, highlighting the formation of the initial normal shock waves.

**Figure 26:**
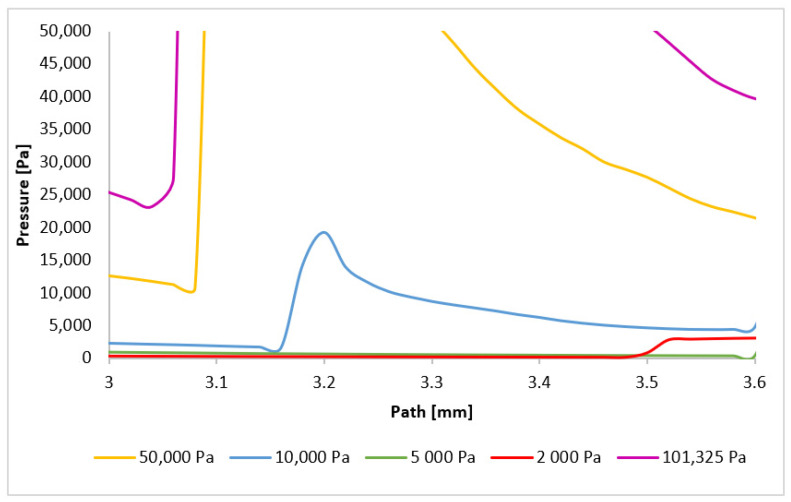
Pressure layout of each variant on the path (axis) with another adjusted scale.

A reversal of the order of normal shock wave formation is observed in the second region (Figure 26). The 101,325 Pa variant exhibits the earliest pressure gradient formation, while the 10,000 Pa variant exhibits the latest. For variants below 10,000 Pa, the dominance of viscous forces over inertial forces prevents sufficient deceleration of the flow. This order reversal for the higher pressure variants can be attributed to the velocity drop in the first region, which influences the phenomena in the second region. A larger decrease to subsonic speeds followed by a more rapid increase leads to the earlier formation of the subsequent normal shock wave. The 101,325 Pa variant experienced the most significant velocity drop in the first region.

**Figure 27:**
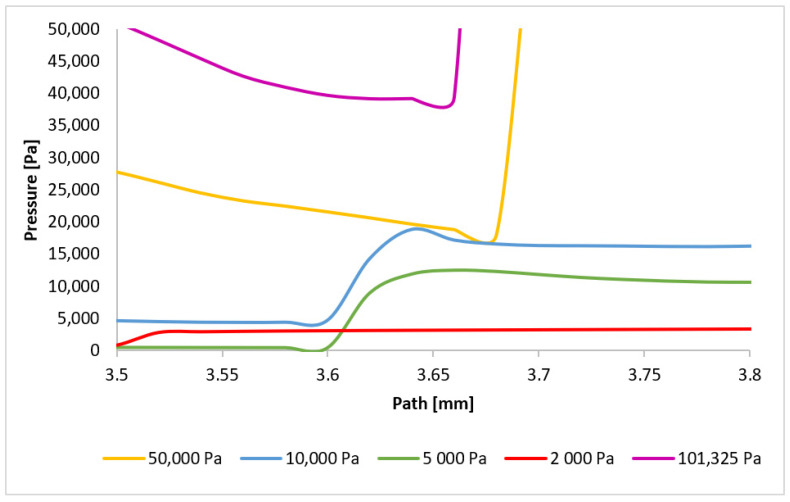
Pressure layout of each variant on the path (axis) with different adjusted scale.

**Figure A2:**
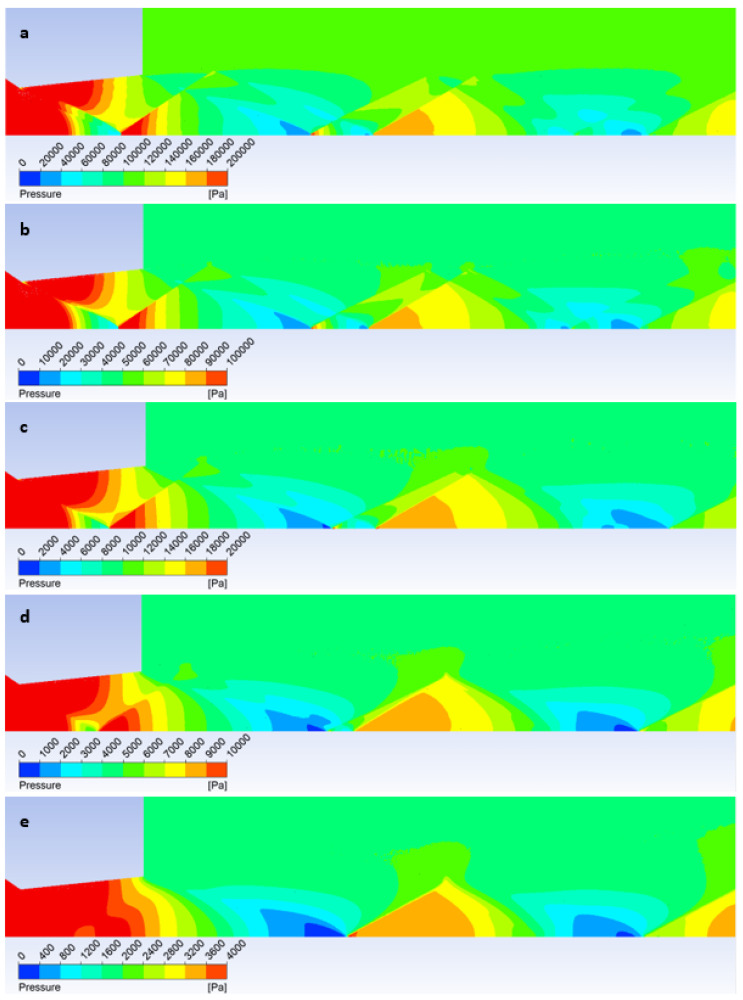
Pressure distribution of each variant: 101,325 Pa (**a**), 50,000 Pa (**b**), 10,000 Pa (**c**), 5000 Pa (**d**), and 2000 Pa (**e**).

Two phenomena become evident in the third region (Figure 27). The trend observed in previous regions, whereby the shock wave forms closer for flow types with more abrupt initial velocity decreases, continues to hold for the 101,325 Pa and 50,000 Pa variants. However, for the 10,000 Pa variant, the reduced influence of inertial forces results in a less pronounced velocity increase following the shock wave, leading to earlier deceleration and shock wave formation. In contrast, for variants with pressures below 10,000 Pa, the flow becomes subsonic for the first time at this distance, resulting in the formation of the initial normal shock wave. The relatively gradual increase in pressure across this shock wave is attributed to its thickness, which is approximately four times the mean free path of the gas molecules [41,42]. The graphical distribution of pressure for each variant can be seen in Appendix A in Figure A2a–e.

**Figure 28:**
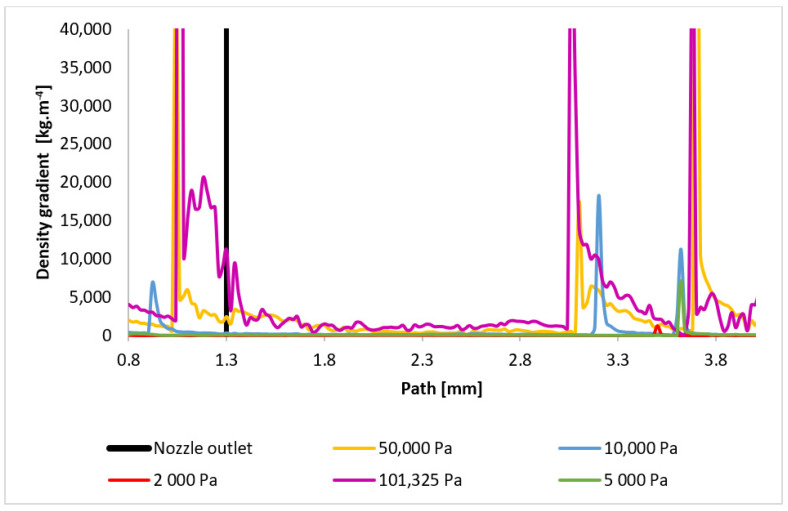
Density gradient layout of each variant on the path (axis).

**Figure A3:**
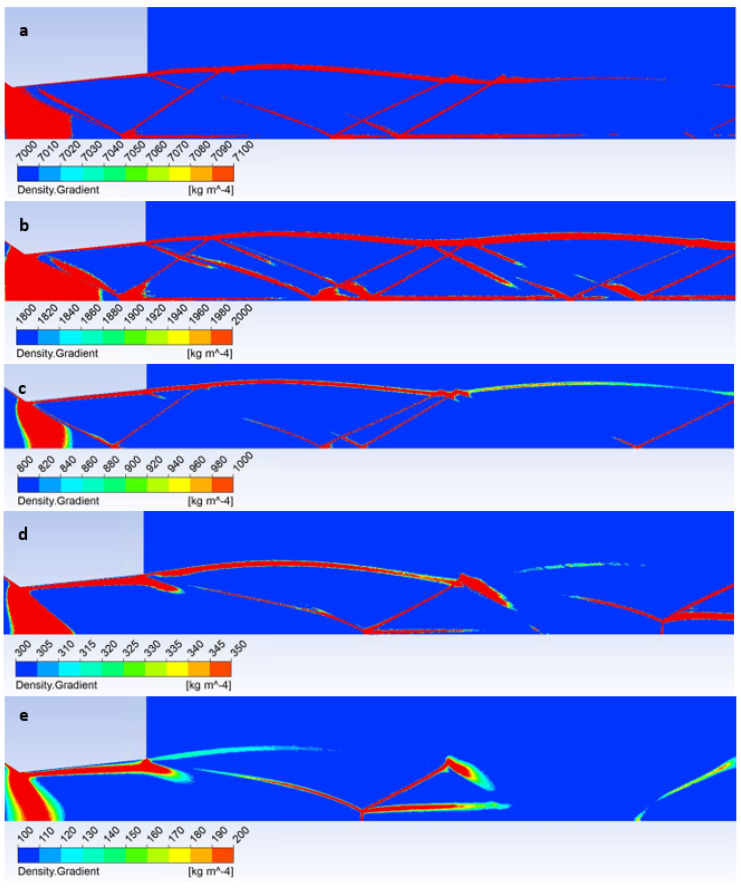
Density gradient distribution of each variant: 101,325 Pa (**a**), 50,000 Pa (**b**), 10,000 Pa (**c**), 5000 Pa (**d**), and 2000 Pa (**e**).

A significant finding from these analyses is that, from a pressure of approximately 10,000 Pa, the flow exhibits a markedly different character due to a significant difference in the ratio of inertial to viscous forces compared to typical atmospheric conditions. This must be considered as differentially pumped chambers in the environmental scanning electron microscope operate within this pressure regime. Figure 28 directly plots the first derivative of the density gradient values, which are used by the Schlieren method. The graphical distribution of the density gradient for each variant can be seen in Appendix A in Figure A3a–e. These values can be used to infer the locations of the assumed normal shock waves for comparison. The results are summarized in Table 7.

**Figure 29:**
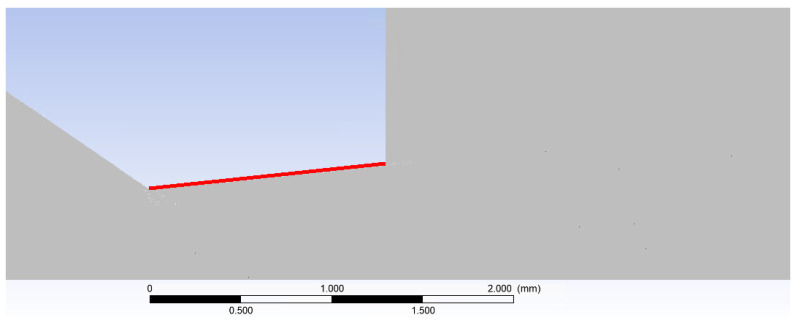
Examined nozzle surface (red line—nozzle wall).

To validate the results, the pressure and temperature distribution on the nozzle wall (Figure 29), which is applicable within the experimental chamber, was analyzed. Six pressure sensors (multirange differential pressure sensors for gases and air DPS 300) and six temperature sensors [43] (custom-made K-type thermocouple with a diameter of 3 mm) were spirally arranged on this nozzle wall [44,45,46].

**Figure 30:**
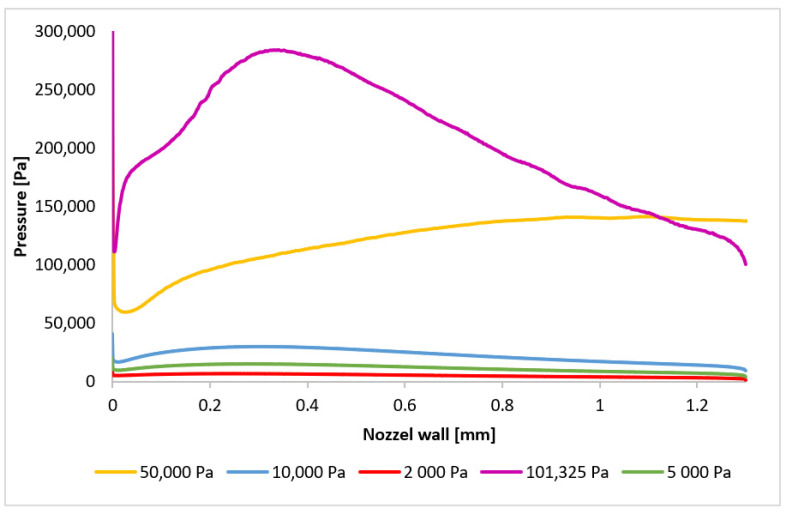
Pressure layout of each variant on the nozzle wall.

**Figure 31:**
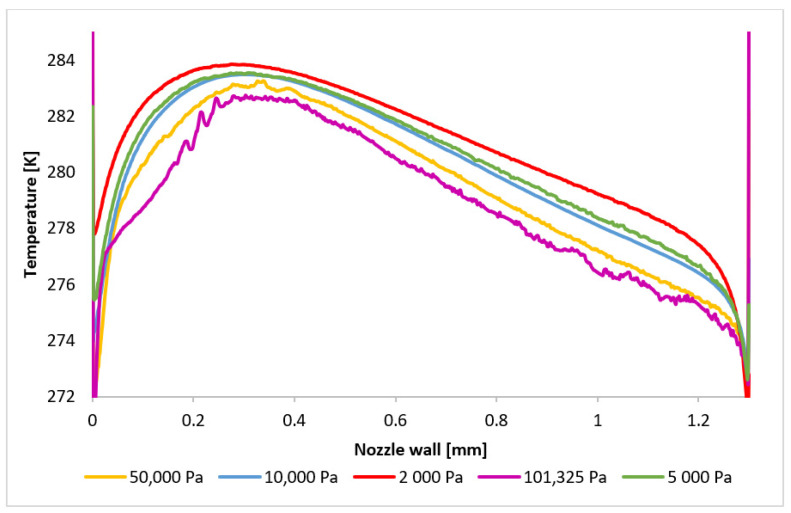
Temperature layout of each variant on the nozzle wall.

The pressure (Figure 30) and temperature (Figure 31) profiles are highly dependent on the velocity profile, providing a comprehensive description of the flow characteristics within the nozzle. These profiles will serve as a benchmark for the comparison between the CFD model and the experimental results.

**Figure 32:**
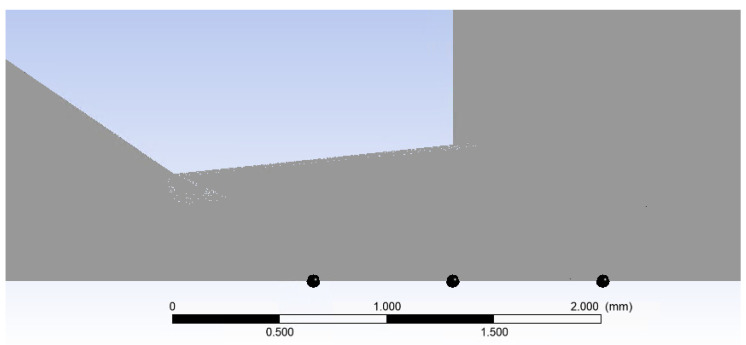
Points for the Reynolds number verification.

Again, the Reynolds number was calculated to determine the flow characteristics in the region where inertial forces and viscous forces interact. Three points were chosen (Figure 32), from which the reference values of the state variables were taken. The results of this evaluation are presented in Table 8.

## 4. Conclusions

This paper presents a comprehensive analysis of supersonic flow characteristics, combining experimental measurements, one-dimensional isentropic flow theory, and CFD simulations. The primary objective is to investigate the shock wave patterns generated by a supersonic flow guided by an aperture and a suitably designed nozzle. The first part of this paper describes the development and validation of an experimental chamber equipped with pressure and temperature sensors. Initial pressure measurements on an overexpanded nozzle were conducted and compared with CFD simulations. This combined approach enabled experimental verification of the CFD results while providing a more detailed description of the flow characteristics than that obtainable through experimental measurements alone. Additionally, preliminary optical method analyses at low pressures at the boundary of continuum mechanics were performed in the experimental chamber and the results will be used for further fine-tuning. The second part of this paper focuses on analyzing the influence of the pressure magnitude on the shock wave arrangements in supersonic flow. The pressure range investigated extended from atmospheric pressure down to low pressures approaching the limits of continuum mechanics. This study was motivated by the application of low pressures in environmental electron microscopy, where the characteristics of supersonic flow during differential pumping significantly impact the electron beam scattering. Initially, the computational dimensions of the nozzle were determined based on one-dimensional isentropic flow theory for a pressure ratio of *p_v_*:*p*_0_ = 0.1. Subsequently, a fine-tuned CFD model was implemented in the Ansys Fluent system to analyze all the variants and evaluate the differences in the shock wave patterns. It is important to note that this study employed a slightly underexpanded nozzle, as the calculated ideal state of the nozzle does not exhibit the formation of normal shock waves. The results demonstrated that with decreasing pressure, and thus with a progressively different ratio of inertial to viscous forces, the location of the first and subsequent normal shock waves varies. Moreover, from a pressure of 10,000 Pa, due to the low inertial forces, a completely different nature of formation of normal shock waves occurs. These results will be used as a foundation for future experiments. Additionally, the results obtained were validated against the one-dimensional isentropic flow theory for the ratios of the state variables across the normal shock wave. These findings provide valuable insights for further research in the field of differentially pumped chambers.

## Figures and Tables

**Table 1:** Comparison of the results obtained from the experimental measuring with the results obtained from the CFD analyses.

	B	C	D
CFD	21,771	13,405	7752
Experiment	21,000	12,800	7600
*ε*	3.5	4.5	1.9

**Table 2:** Results of the first cell size *y* at the given points.

		Aperture				
Position	*y* [mm]	*µ* [10^−5^ Pa·s]	*ρ* [kg·m^−3^]	*V_max_* [m·s^−1^]	*D* [mm]	*T* [K]
Aperture	0.0022	1.56	0.73	297.7	2	254.5
Nozzle	0.005	0.803	0.075	602.1	3.88	120.4

**Table 3:** Results of the Reynolds number at the given points.

Point on Path [mm]	Char. Dimension [m]	Mean Velocity [m·s^−1^]	Density [kg·m^−3^]	Dyn. Viscosity [Pa·s]	Temperature [K]	Re Number [-]
0	0.002	148.85	0.73	1.56 × 10^−5^	254.5	13,930.83
2.5	0.0027	142.55	0.241	1.57 × 10^−5^	255.5	5908.11
5	0.0035	287.4	0.095	8.98 × 10^−5^	135.2	10,641.48
7.5	0.08	118.3	0.196	1.63 × 10^−5^	268.5	111,730.47

**Table 4:** Specific pressure ratios for further CFD analyses and their respective marking.

Variant (Further Marked)	*p*_0_ (Input Pressure) [Pa]	*p_v_* (Output Pressure) [Pa]
101,325 Pa	1,013,250	101,325
50,000 Pa	500,000	50,000
10,000 Pa	100,000	10,000
5000 Pa	50,000	5000
2000 Pa	20,000	2000

**Table 5:** Values calculated from Equations (1)–(6).

	Labeling	Unit	Theory
Mach number	*M_v_*	[-]	2.16
Speed of sound in the chamber for *T*_0_ = 24 °C	*a*_0_	[m·s^−1^]	345.5
Outlet speed of sound	*a_v_*	[m·s^−1^]	248.5
Outlet velocity	*v_v_*	[m·s^−1^]	536.8
Outlet temperature	*T_v_*	[K]	153.7
Outlet cross-section	*A_v_*	[mm^2^]	1.39

**Table 6:** Comparison of the results from one-dimensional flow theory with the results obtained from the CFD simulations.

	Labeling	Unit	Theory	Point	Line
Mach number	*M_v_*	[-]	2.16	2	2.1
Speed of sound in the chamber for *T*_0_ = 24 °C	*a*_0_	[m·s^−1^]	345.5	-	-
Outlet speed of sound	*a_v_*	[m·s^−1^]	248.5	-	-
Outlet velocity	*v_v_*	[m·s^−1^]	536.8	530.9	530
Outlet temperature	*T_v_*	[K]	153.7	160	157
Outlet cross-section	*A_v_*	[mm^2^]	1.39	-	-
**Variant 101,325 Pa**					
Outlet static pressure	*p_v_*	[Pa]	101,325	250,906	102,324
Outlet density	*ρ_v_*	[kg·m^−3^]	2.29	2.9	2.2
**Variant 50,000 Pa**					
Outlet static pressure	*p_v_*	[Pa]	50,000	130,398	50,906
Outlet density	*ρ_v_*	[kg·m^−3^]	1.13	1.48	1.1
**Variant 10,000 Pa**					
Outlet static pressure	*p_v_*	[Pa]	10,000	13,924	10,569
Outlet density	*ρ_v_*	[kg·m^−3^]	0.226	0.29	0.227
**Variant 5000 Pa**					
Outlet static pressure	*p_v_*	[Pa]	5000	6580	5495
Outlet density	*ρ_v_*	[kg·m^−3^]	0.113	0.126	0.116
**Variant 2000 Pa**					
Outlet static pressure	*p_v_*	[Pa]	2000	2506	2288
Outlet density	*ρ_v_*	[kg·m^−3^]	0.045	0.051	0.049

**Table 7:** Distance of the shock wave from the aperture.

		Shock Wave Position	
Variant	1 [mm]	2 [mm]	3 [mm]
101,325 Pa	1.06	3.06	3.68
50,000 Pa	1.04	3.1	3.7
10,000 Pa	0.92	3.2	3.62
5000 Pa	-	-	3.62
2000 Pa	-	-	3.5

**Table 8:** Results of the Reynolds number at the given points.

Variant	Point	Char. Dimension [m]	Mean Velocity [m·s^−1^]	Density [kg·m^−3^]	Dyn. Viscosity [Pa·s]	Temperature [K]	Re Number [-]
101,325 Pa	1	0.00113	258.5	2.74	1.09 × 10^−5^	167	73,428.23
	2	0.00127	186	2.91	1.43 × 10^−5^	229	41,935.86
	3	0.08	246	1.73	1.16 × 10^−5^	178	2,935,034.48
50,000 Pa	1	0.00113	259	1.35	1.09 × 10^−5^	167	36,248.12
	2	0.00127	182.5	1.36	1.45 × 10^−5^	232	21,738.9
	3	0.08	241	0.84	1.18 × 10^−5^	181.6	1,372,474.58
10,000 Pa	1	0.00113	259	0.27	1.1 × 10^−5^	168	7203.36
	2	0.00127	239.5	0.323	1.2 × 10^−5^	186	8180.29
	3	0.08	272.5	0.208	1.01 × 10^−5^	154	448,950.5
5000 Pa	1	0.00113	258.5	0.137	1.1 × 10^−5^	169	3624.85
	2	0.00127	245	0.165	1.18 × 10^−5^	182	4350.83
	3	0.08	273	0.109	1.01 × 10^−5^	154	234,769.23
2000 Pa	1	0.00113	242	0.0685	1.196 × 10^−5^	184	1574.12
	2	0.00127	243.5	0.067	1.18 × 10^−5^	183	1755.88
	3	0.08	269.5	0.046	1.03 × 10^−5^	157	96,287.38

## Data Availability

The data presented in this study are available on request from the corresponding author.
